# Socioeconomic inequalities in curative healthcare-seeking for children under five before and after the free healthcare initiative in Sierra Leone: analysis of population-based survey data

**DOI:** 10.1186/s12939-021-01474-7

**Published:** 2021-05-21

**Authors:** Joel D. Bognini, Sekou Samadoulougou, Mady Ouedraogo, Tiga David Kangoye, Carine Van Malderen, Halidou Tinto, Fati Kirakoya-Samadoulougou

**Affiliations:** 1 Unité de Recherche Clinique de Nanoro, Institut de Recherche en Sciences de la Santé, Centre National de la Recherche Scientifique et Technologique, 42 Avenue Kumda-Yonre, Kadiogo 11 BP 218 Ouagadougou CMS 11, Ouagadougou, Burkina Faso; 2 Centre for Research on Planning and Development (CRAD), Laval University, Quebec, G1V 0A6 Canada; 3 Evaluation Platform on Obesity Prevention, Quebec Heart and Lung Institute, Quebec, G1V 4G5 Canada; 4 Centre de Recherche en Epidémiologie, Biostatistiques et Recherche Clinique, Ecole de Santé Publique, Université Libre de Bruxelles, Bruxelles, Belgium; 5 Institut National de la Statistique et de la Démographie [INSD], Ouagadougou, Burkina Faso; 6 Centre National de Recherche et de Formation sur le Paludisme (CNRFP), 01 BP 2208, Ouagadougou, 101 Burkina Faso; 7 Institut de Recherche Santé et Société (IRSS), Université catholique de Louvain, Bruxelles, Belgium; 8 Clinical Research Unit of Nanoro, Institute for Research in Health Sciences, National Center for Scientific and Technological Research, 42, Avenue Kumda-Yoore, BP 218 Ouagadougou CMS 11, Ouagadougou, Burkina Faso; 9 Centre de Recherche en Epidémiologie, Biostatistiques et Recherche Clinique, École de Santé Publique, Université Libre de Bruxelles, Brussels (ULB), Route de Lennik, 808, B-1070 Brussels, Belgium

**Keywords:** Socioeconomic inequalities, Healthcare utilization, Children under five, Sierra Leone

## Abstract

**Background:**

Socioeconomic inequalities between and within countries lead to disparities in the use of health services. These disparities could lead to child mortality in children under 5 years by depriving them of healthcare. Therefore, initiatives to remove healthcare fees such as the Free Healthcare Initiative (FHCI) adopted in Sierra Leone can contribute to reducing these inequities in healthcare-seeking for children. This study aimed to assess the socioeconomic inequalities in healthcare-seeking for children under 5 years of age before and after the implementation of the FHCI.

**Methods:**

Data were included on 1207, 2815, 1633, and 1476 children under 5 years of age with fever from the 2008, 2013, 2016, and 2019 nationwide surveys, respectively. Concentration curves were drawn for the period before (2008) and after (2013–2019) the implementation of the FHCI to assess socioeconomic inequalities in healthcare-seeking. Finally, Erreyger’s corrected concentration indices were calculated to understand the magnitude of these inequalities.

**Results:**

Before the implementation of the FHCI, there were inequalities in healthcare-seeking for children under five (Erreyger’s corrected concentration index (CI) = 0.168, standard error (SE) = 0.049; *p* < 0.001) in favor of the wealthy households. These inequalities decreased after the implementation of the FHCI (CI = 0.061, SE = 0.033; *p* = 0.06 in 2013, CI = 0.039, SE = 0.04; *p* = 0.32 in 2016, and CI = − 0.0005, SE = 0.362; *p* = 0.98 in 2019). Furthermore, before the implementation of the FHCI, a significant pro-rich inequality in the districts of Kenema (CI = 0.117, SE = 0.168, *p* = 0.021), Kono (CI = 0.175, SE = 0.078, *p* = 0.028) and Western Area Urban (CI = 0.070, SE = 0.032, *p* = 0.031) has been observed. After the implementation of the FHCI in 2019, these disparities were reduced, 11 of the 14 districts had a CI around the value of equality, and only in 2 districts the pro-rich inequality were significant (Western Area Urban (CI = 0.035, SE = 0.016, *p* = 0.039) and Western Area Rural (CI = 0.066, SE = 0.030, *p* = 0.027)).

**Conclusion:**

The results of this study demonstrated that socio-economic inequalities in healthcare-seeking for children have been considerably reduced after the FHCI in Sierra Leone. To further reduce these inequalities, policy actions can focus on the increase of availability of health services in the districts where the healthcare-seeking remained pro-rich.

## Background

Notable progress has been made in reducing child mortality over the past 3 decades but improving child survival remains an urgent concern, especially in sub-Saharan Africa (SSA). Global estimates show that under-5 mortality declined in by 59%, from 93 deaths per 1000 live births in 1990 to 38 in 2019 [1]. The highest rates are still seen in SSA where more than 50% of the estimated 5.2 million children under-5 died in 2019 [1].

In SSA, most under-5 deaths are preventable with effective low-cost interventions but prevailing inequalities between subpopulations within countries in this region [2] pose a major challenge in expanding access to such interventions particularly in disadvantaged groups and areas with poor access to health services [3]. The poorest subpopulations thus remain lagging compared to their richest counterparts [3, 4].

Disparities in access to healthcare between the least and most deprived groups within a country are well established [5, 6], and factors related to socioeconomic status have been shown to exert varied effects on the variability of under-5 mortality in SSA [7]. Therefore, interventions within-country level plays a vital role in eliminating health inequalities. Recent evidence demonstrates that countries that have attained high coverage of maternal, newborn and child health interventions will also show reduced socio-economic inequalities in these coverage levels [6, 8–10].

In Sierra Leone, access to healthcare has been problematic over the past 2 decades especially for the vulnerable populations due to health system constraints from civil wars (1991–2002) [11] and waves of disease epidemics (Cholera and Ebola) [12, 13]. This was evident via the high rate (30%) of children under-5 who did not have access to healthcare in 2013 [14] and a slow decline in under-5 mortality from 120 deaths per 1000 live births in 2015 to 102 in 2020 [15]. In response, the government implemented the Free Healthcare Initiative (FHCI) for children under-5, pregnant women and lactating mothers in 2010 to reduce the inequalities in terms of access to healthcare and high mortality rate [16].

Since its implementation, the utilization of health services, particularly antenatal and postnatal services increased [17–19]. Antenatal care visits and institutional delivery increased by 25% from 2008 to 2013 [19], and the proportion of healthcare-seeking for febrile illness in children under-5 also increased from 62% in 2013 to 71% in 2016 [18]. However, this increase in accessibility to healthcare was unevenly distributed between wealth levels, regions, and types of places of residence [19].

**Fig. 1:**
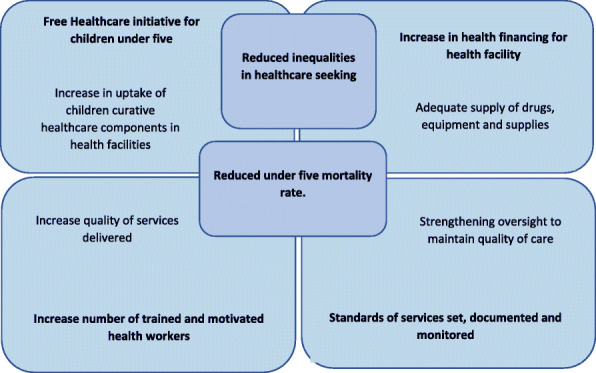
Theoretical framework of free healthcare initiative adapted from Manso et al. [20]

The theoretical framework in Fig. 1 below explains how the FHCI is expected to work to increase healthcare-seeking for febrile children and lead to the reduction of the mortality of children under five. If health services are available and accessible in terms of cost and distance, and the quality of these services improved and sustained by motivated, competent workers, then socioeconomic inequalities will decrease. Consequently, more caregivers will use the free under-five mortality will rate.

To date, no study has assessed the impact of the FHCI on the socioeconomic inequalities in access to curative healthcare for children under five in Sierra Leone. Using data collected from 4 cycles of the Sierra Leone Demographic Health Surveys (DHS 2008, 2013, 2016 and 2019), this study sort to assess the inequalities in access to healthcare for children under five in terms of quantity and magnitude before and after the implementation of the FHCI in Sierra Leone.

Such additional knowledge could be used for designing finer investigations to uncover and address the drivers of the inequalities persisting where the FHCI aimed at flattening them.

## Methodology

### Setting

Sierra Leone is a West African country located on the southwest coast, with a surface area of 71,740 km^2^ and an estimated population of 7,396,000 in 2016 [21]. The population is generally young, with about 63% of the total population aged less than 25 years, 17% representing children under 5 years [22]. Like other SSA countries, it has a tropical climate with two seasons and vegetation ranging from the savannah in the North to the rainforest in the South.

For these surveys, the country was divided into four administrative regions (the northern, eastern, southern, and western regions), and each region further subdivided into districts (total of 14 districts) in the country.

The health system in Sierra Leone is organized into three levels. The primary level, which is made up of peripheral health units (PHUs), of which 229 are community health centers (CHCs), 386 community health posts (CHPs), and 559 maternal and child health posts (MCHPs). The secondary level which consists of 21 district hospitals [23], and the tertiary level which includes regional and specialized hospitals. There are six teaching hospitals and several private clinics and hospitals spread across the 14 districts of the country. The distribution of the wealth index is not equal between the rural and urban areas. Approximately 61% of the population in the urban area are richest and in the rural area, 28% are poorest [24], while the inequality-adjusted human development index was low at 0.266 in 2017 [25].

### Data source

This study used data from the 2008, 2013, and 2019 Sierra Leone Demographic and Health Surveys (DHS) and the 2016 Malaria Indicator Survey (MIS). These are national representative household surveys in which women aged 15–49 years were interviewed. Details of the complete description of the interview methods are available elsewhere [24, 26]. The current study focused on febrile children under 5 years of age whose caregivers sought healthcare in the 2 weeks preceding each of the surveys.

### Variables

#### Outcome measure

The outcome variable is healthcare-seeking, which is the proportion of under-five children with fever in the 2 weeks preceding the surveys, whose caregivers sought care in public or private (coded 1) health centers or traditional/other (coded 0).

#### Socioeconomic variable

Household wealth index is used as the main socioeconomic variable in this study. The wealth index is a composite indicator of inequalities in household characteristics and was categorized (richest, richer, middle, poor, and poorest) based on the household’s ownership of consumer goods; dwelling characteristics; type of drinking water source; toilet facilities; and other characteristics that relate to a household’s socioeconomic status. The detailed construction and explanation of this variable has been previously described elsewhere [24, 26, 27].

#### Other variables

The independent variables were chosen based on similar studies [19, 28] and included sociodemographic variables and the determinants of healthcare-seeking for febrile children. The respondent’s age which was recorded as 15 to 59 years was re-categorized (15–24 years for younger mothers, 25–34 years for middle aged mothers, and ≥ 35 years for old mothers). Also, the mother’s level of education; attended school? Yes = 1 and No = 0, and the school level which was coded as primary = 0, junior secondary = 1, senior secondary = 2, vocational / commercial / nursing technical / teaching = 3 and higher = 4 was re-categorized (no formal education, primary education, and secondary education or higher). The total number of children born in the household recorded 0 to 9 and more was categorized as 1–2, 3–4, and ≥ 5 children. The sex of the head of the household (male = 1 and female = 0); the age of the head of the household (15–24, 25–34, and ≥ 35 years); the regions (east, north, south, and west); the districts of residence (Kailahum, Kenema, Kono, Bombali, Kambia, Koinadugu, Port Loko, Tonkolili, Bo, Bonthe, Moyamba, Pujehun, Western Area Rural, and Western Area Urban); the types of places of residence (coded rural = 0 or urban = 1) were also used. Child age recorded as 0 to 59 months was categorized (< 12, 12–35, and 36–59 months), and together with the sex of the child (male = 1 and female = 0), child’s household size (recorded 0 to 9 and more); and the type of facility where healthcare was sought (public, private, traditional, or other) were also selected as independent variables.

#### Sampling method

A two-stage cluster sampling method was used in all the 4 population-based surveys. The country was sub-divided into enumeration areas (EAs), and each EA included several households. In the first stage, EAs were selected with stratified probability proportional to sample size, and the place of residence (urban or rural) was used to stratify the EAs. In the second stage, households were selected using systematic random sampling from the EAs. The sampling frames were developed based on the 2004 census for the 2008 and 2013 surveys and on the 2015 census for the 2016 and 2019 surveys [22, 24, 26].

#### Statistical methods

Statistical analyses were performed using Stata version 15.0. The characteristics of the children’s caregivers and those of the febrile children whose sought healthcare were described. For the analysis of wealth related inequalities in healthcare seeking, participants were grouped (according to their socioeconomic status) into wealth quintiles, as follows: Poorest (1st quintile), poorer (2nd quintile), middle (3rd quintile), richer (4th quintile), and richest (5th quintile). Analyses were performed separately for each survey to evaluate the proportion of caregivers seeking care for their febrile children. The descriptive analyses were weighted for probability sampling and adjusted for stratification and clustering. An alpha 0.05 was set for statistically significant difference between compared groups.

#### Measuring socioeconomic inequalities

The concentration curves and the concentration index were used to assess socioeconomic inequalities in the use of healthcare at the country level. At the district level, the concentration index was used to estimate the socioeconomic inequalities in the healthcare-seeking for children under five. The concentration curves were used to examine the trend in the pattern of the socioeconomic inequalities in healthcare-seeking, while the concentration index was used to assess the magnitude of the inequalities in healthcare-seeking. The use of the concentration curve and index in the quantification of the degree of the socioeconomic-related inequality in the health-state indicators is well documented [29–32].

#### Concentration curves

The concentration curves (Lorenz curve) were built using two keys’ variables: The wealth index and the healthcare-seeking for febrile children under five. The concentration curves represent a plot of the cumulative percentage of caregivers seeking care (y-axis) against the cumulative percentage of the households, ranked by the wealth index, beginning with the poorest, and ending with the richest (x-axis) [33].

The concentration curves were compared to the line of equality which is the 45-degree line running from the bottom left-hand corner to the upper right-hand corner, indicating the absence of inequalities in healthcare-seeking between the households ranked in the wealth quintiles. When the percentage of caregivers seeking care takes higher values among poorer households, the concentration curves lie above the line of equality. On the contrary, when it takes lower values among poorer households, the concentration curves lie below the line of equality. The farther the curves are above the line of equality, the more concentrated the health variable is among the poor [33].

#### Concentration index

The concentration index estimates the magnitude of wealth-related inequalities in healthcare-seeking. The formula of the concentration index is as follows:

$$ C=\frac{2}{\mu}\operatorname{cov}\left(h,r\right) $$

where *h* represents healthcare-seeking, *μ* represents its mean, *r* is the fractional rank of an individual in the wealth index distribution, and *cov* is the covariance between care-seeking and the fractional rank of the wealth index [33]. The concentration index is calculated as twice the area between the curve and the line of equality. It’s also bounded between − 1 and 1.

In absence of wealth-related inequalities, the concentration index is zero. The concentration index takes a negative value when the curve lies above the line of equality, indicating a disproportionate concentration of healthcare-seeking among the poor. It takes a positive value when it lies below the line of equality, indicating a concentration of healthcare-seeking among the richer [33]. Given that the bounds of the C of a binary health indicator depend on the mean of this indicator which varied according to the periods studied, the Erreygers’ normalization option was selected in Stata. QGIS 3.12 software was used to map the concentration indices for healthcare-seeking for children under five at the health district level [34].

## Results

In total, 1207 (21.5% of the total of children in 2008), 2815 (23.6% of the total of children in 2013), 1633 (26.3% of the total of children in 2016) and 1476 (15% of the total of children in 2019) children under five with a history of fever were included from the 2008, 2013, 2016 and 2019 surveys, respectively.

### Characteristics of caregivers and children and healthcare-seeking for fever

Table 1 illustrates the healthcare-seeking for children under five by caregivers and children characteristics. Close to half (more than 45%) of the caregivers aged between 25 and 34 years sought care for fever in all four surveys. Similarly, about 50% of those who solicited care for fever came from large families (6–10 members) whilst over a third (more than 30%) had 3 or more children. Three in four (about 75%) caretakers belonged to households headed by middle-aged or older men. About two thirds (60%) were uneducated, came from rural areas and resided mainly in the Northern and Southern. Close to half (more than 45%) of the children were aged 12–35 months.

**Table 1:** Healthcare-seeking for children under five by caregivers and children characteristics

Characteristics of the population	2008	2013	2016	2019
	n (%)	n (%)	n (%)	n (%)
**Caregivers**				
**Age of mother (years)**				
15–24	190 (28.4)	604 (28.8)	400 (34.2)	339 (28.3)
25–34	318 (50.2)	971 (47.1)	555 (46.3)	533 (48.1)
≥ 35	121 (21.4)	499 (24.1)	218 (19.5)	249 (23.6)
**Number of family members**				
1–5	183 (26.0)	608 (31.0)	374 (30.4)	424 (37.6)
6–10	356 (58.8)	1114 (52.4)	609 (52.5)	546 (48.8)
> 10	90 (15.2)	352 (16.6)	190 (17.1)	152 (13.6)
**Total number of children born**				
1–2	251 (37.7)	702 (33.2)	369 (33.2)	420 (36.8)
3–4	195 (30.4)	661 (32.5)	358 (31.2)	368 (31.4)
≥ 5	183 (31.9)	711 (34.3)	446 (35.6)	333 (31.8)
**Mother’s education level**				
None	388 (64.9)	1381 (65.8)	688 (57.8)	582 (51.7)
Primary	108 (16.1)	309 (14.9)	188 (14.9)	194 (18.0)
Secondary or higher	133 (19.0)	384 (19.3)	297 (27.3)	345 (30.3)
**Gender of the household head**				
Male	491 (78.7)	1530 (74.7)	895 (76.1)	884 (77.8)
Female	138 (21.3)	544 (25.3)	278 (23.9)	237 (22.2)
**Age of the household head (years)**				
15–24	23 (3.3)	73 (3.7)	34 (2.8)	45 (3.8)
25–34	156 (22.5)	514 (24.8)	262 (20.5)	255 (22.3)
≥ 35	450 (74.2)	1487 (71.5)	877 (76.7)	821 (73.9)
**Place of residence**				
Urban	270 (35.1)	669 (27.8)	323 (34.1)	345 (34.8)
Rural	359 (64.9)	1405 (72.2)	850 (65.9)	776 (65.2)
**Region**				
East	188 (12.5)	472 (26.0)	254 (25.3)	268 (26.0)
North	184 (40.6)	891 (39.4)	447 (37.6)	173 (12.6)
South	170 (24.5)	525 (23.1)	388 (26.8)	218 (18.9)
West	157 (22.4)	186 (11.5)	84 (10.3)	462 (42.5)
**CHILDREN**				
**Gender of the child**				
Male	326 (50.8)	1055 (50.7)	595 (49.4)	563 (50.0)
Female	303 (49.2)	1019 (49.3)	578 (50.6)	558 (50.0)
**Age (months)**				
< 12	88 (31.7)	235 (22.7)	266 (23.0)	125 (20.9)
12–35	129 (45.9)	465 (46.8)	558 (45.9)	289 (47.3)
36–59	66 (22.4)	317 (30.5)	349 (31.1)	188 (32.8)

**Fig. 2:**
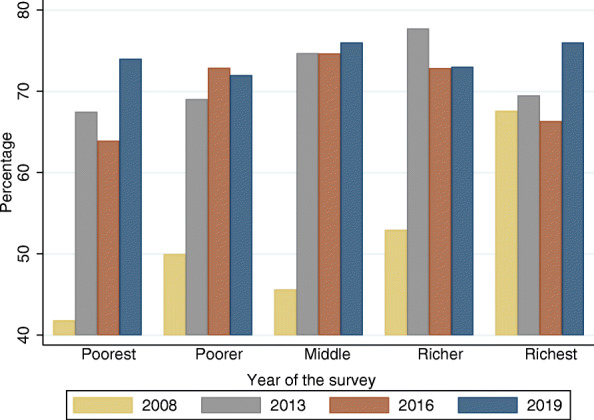
Distribution of healthcare-seeking for children under five by wealth index and by year

Healthcare-seeking for children under five with fever increased from 51% in 2008 to 71.6, 70, and 74% in 2013, 2016, and 2019, respectively. Figure 2 shows the healthcare-seeking behavior for children under five by the wealth index. Healthcare-seeking was increased between 2008 and 2019 in each socioeconomic status, with the most increase observed among poorest population.

### Socioeconomic inequalities in healthcare-seeking

**Fig. 3:**
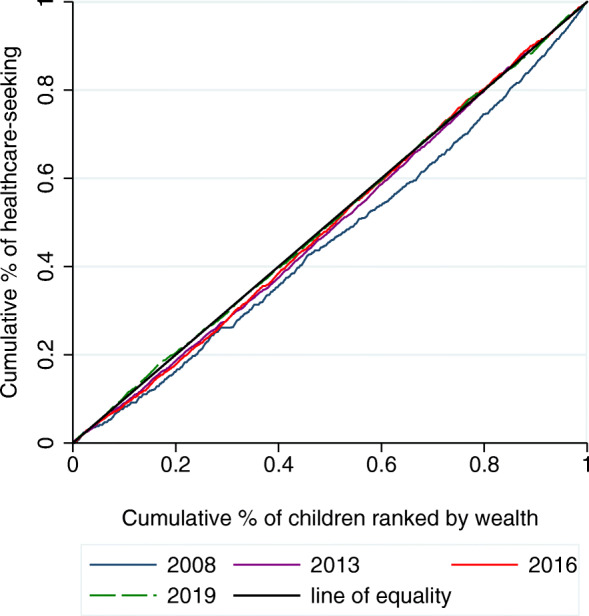
Concentration curves for healthcare-seeking for children under five, 2008–2019

Figure 3 shows the concentration curves of healthcare-seeking for children under five with fever before (2008) and after (2013, 2016, and 2019) the implementation of the FHCI. In 2008, before the initiation of the FHCI, socioeconomic-related inequalities in healthcare-seeking favored the rich households, meaning that healthcare-seeking was concentrated among the wealthy households. Following the implementation of the FHCI, the inequalities declined progressively up to the line of equality in 2019.

**Fig. 4:**
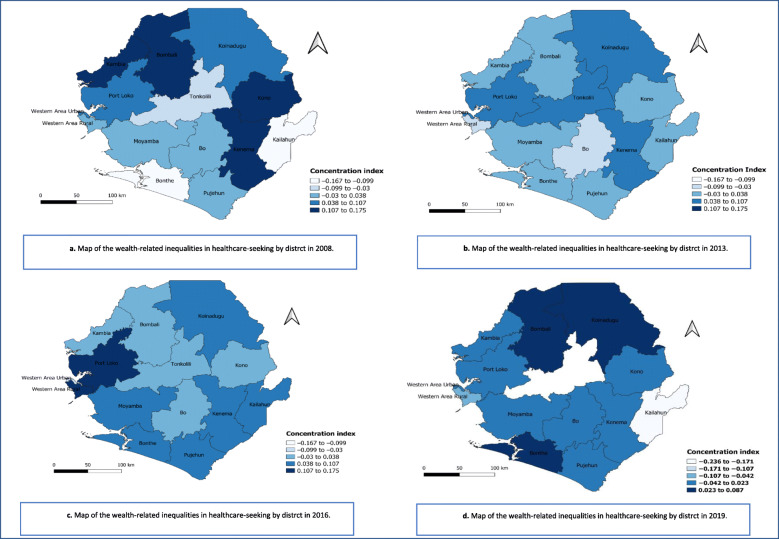
Maps of the wealth-related inequalities in healthcare-seeking at the district level, 2008–2019

The Fig. 4 shows the wealth-related inequalities in healthcare-seeing for febrile children under five in the four surveys at the district level. Healthcare-seeking was pro-rich in seven districts in 2008, while in 2019 there were three pro-rich districts.

In 2008, a significant pro-rich inequality in the districts of Kenema (CI = 0.117, SE = 0.168, *p* = 0.021), Kono (CI = 0.175, SE = 0.078, *p* = 0.028) and Western Area Urban (CI = 0.070, SE = 0.032, *p* = 0.031) has been observed (Fig. 4a).

In 2013, healthcare-seeking for febrile children was significantly pro-rich in the districts of Kenema (CI = 0.073, SE = 0.018, *p* < 0.001), Bombali (CI = 0.037, SE = 0.016, *p* = 0.024), Koinadugu (CI = 0.068, SE = 0.023, *p* = 0.004), Port Loko (CI = 0.083, SE = 0.027, *p* = 0.003), and Bo (CI = 0.081, SE = 0.24, p < 0.001), (Fig. 4b).

In 2016, a significant pro-rich inequality was observed in the districts of Port Loko (CI = 0.171, SE = 0.062, *p* = 0.008), Bonthe (CI = 0.066, SE = 0.030, *p* = 0.030), Pujehun (CI = 0.053, SE = 0.017, p = 0.004), and in Western Area Rural (CI = 0.174, SE = 0.056, p = 0.003) (Fig. 4c).

In 2019, these disparities 11 of the 14 districts had a CI around the value of equality, and in 2 districts the pro-rich inequality were significant (Western urban Area (CI = 0.035, SE = 0.016, *p* = 0.039) and Western Area Rural (CI = 0.066, SE = 0.030, *p* = 0.027) (Fig. 4d).

### Concentration index of healthcare-seeking for children under five with fever

To better appreciate the magnitude of the inequalities, the authors computed the concentration index as reported in Table 2.

**Table 2:** The concentration index for healthcare-seeking for children under five by year

Outcome	2008			2013			2016			2019		
	CI	SE	***p***-Value	CI	SE	***p***-Value	CI	SE	***p***-Value	CI	SE	***p***-Value
**Healthcare-seeking**	0.168	0.049	< 0.001	0.061	0.033	0.061	0.039	0.040	0.32	−0.0005	0.362	0.98

*CI* Erreygers’ corrected concentration index, *SE* standard error

The positive concentration index in 2008 suggests that healthcare was more accessible to rich households. A decrease in the magnitude of the inequalities was noted after the implementation of the FHCI (2013–2019), as indicated by a 2019 concentration index, close to zero (− 0.0005).

## Discussion

This study aimed to assess socioeconomic inequalities in access to healthcare among under-five children in Sierra Leone with febrile illness before and after the implementation of the FHCI. This study showed evidence of wealth-related inequalities in healthcare-seeking for febrile children under five in favor of rich households before the implementation of the FHCI at the country level. However, these inequalities decreased following the implementation of the policy. It also highlights a difference of these inequalities in healthcare-seeking for children under-five between districts before and after the FHCI.

The study results are consistent with previous reports on wealth-related inequalities in favor of wealthy households [19, 28, 35]. The use of healthcare has a cost for societies [36, 37], and making access to health services possible for everyone by the policy makers remains a major challenge for developing countries. In 2008, healthcare costs were one of the major barriers to accessing healthcare [38], as evidenced by a positive concentration index of around 0.168, favoring the wealthy households. After the initiation of free healthcare for children under the age of five, a notable decrease in the inequalities in healthcare-seeking from 0.168 in 2008 to 0.04 in 2016, and − 0.0005 in 2019 was observed. This could be explained by a reduction in the barriers represented by the costs of care, thereby allowing access to healthcare for a fringe of children under five belonging to poor households. Moreover, this decrease could be explained by the reinforcement of resources in the health domain such as the increased density of health workers between 2008 and 2016. Indeed, the density of physicians increased from 0.016 to 0.025 per 1000 people, and that of midwives and nurses increased from 0.162 to 0.224 per 1000 people within the same period [39, 40]. This increase in the density of health staff could explain the fact that, in some districts, inequalities in healthcare-seeking showed a considerable reduction, which led to a reversal in healthcare-seeking in favor of vulnerable social strata.

In 2019 compared to 2008, the number of districts with an equal access to healthcare were high, this is consistent with reports on the sustainability and the equitable delivery of the FHCI in the country [16, 17, 41]. This suggest that efforts made to cover all the country with the health services are perceptible in the district level. With the difficulties experienced in the country by the health system, many non-governmental organizations are working to make available healthcare for vulnerable population by providing medical supplies and training health workers [42, 43]. These interventions conjugated with the FHCI have seen the reduction of the socioeconomic inequalities in healthcare-seeking for children under-five years of age by reducing the number of districts where healthcare-seeking were pro-rich.

It remains districts where healthcare-seeking is pro-rich in the context of free healthcare in 2019, namely Port Loko, Bonthe, Pujehun and Western Area Rural. Bonthe for example is made of mainland, and many isolated islands, and then are the most remote, hardest-to-reach areas of the country. It is part of district hardly experienced by Ebola outbreak and many health facilities were closed, reducing the accessibility to the healthcare [44, 45], maintaining the pro-rich inequalities in healthcare-seeking, as for Pujehun, it is one of the country’s poorest and least developed districts. This limited the access of population to the health services, because there is one hospital for a population of over 335,000 [46].

Port Loko the most populous, and Western Area Rural districts were affected by the Ebola outbreak and considered as the “hotspot” of the outbreak. In additional, over 30% of the population are food insecure resulting to a slow progression of the level of social services and economic recovery since the end of the outbreak [47, 48]. The difficulties to cover needs of the districts in terms of health services led to the remaining pro-rich in the healthcare-seeking for children under five.

The lack of health resources made healthcare-seeking for children under five more accessible to rich households despite the free health care initiative in these districts.

One of the strengths of this paper is that the study used nationally representative data that allows the generalization of findings to the entire country. Moreover, the study presents, for the first-time, the wealth-related inequalities in healthcare-seeking for children under 5 years in Sierra Leone. However, this study, is subject to some limitations. Even if, the decrease in the socioeconomic inequalities coincided with the implementation of the FHCI, we cannot make causal interpretation due to the cross-sectional nature of the data. Some factors that may affect healthcare-seeking such as the quality of health services, the availability of supplies, and the differences in the sociocultural conception of illness and its etiology may have contributed to the socio-economic inequalities.

## Conclusion

The current study pointed out that the inequalities in healthcare-seeking in Sierra Leone, were globally in favor of wealthy households before the FHCI. After the FHCI, these inequalities were reduced, though with some observable residual inequalities still in favor of wealthy households in two districts in 2019. Public health programs could be adjusted to better address drivers other than financial barriers so that inequalities in healthcare-seeking for children in Sierra Leone are reduced further.

## Data Availability

The datasets analyzed during the current study are available from the Demographic and Health Surveys program repository and are accessible after written request: https://dhsprogram.com/data.

## Abbreviations

CHC: Community healthcare
CHP: Community health post
CI: Concentration index
DHS: Demographic and Health Surveys
EAs: Enumeration areas
FHCI: Free Healthcare Initiative
ICF: International Coach Federation
MCHP: Maternal and child health post
PHC: Primary healthcare
PHU: Peripheral health unit
SDGs: Sustainable Development Goals
SE: Standard error
SSA: Sub-Sahara Africa
WHO: World Health Organization
